# Effect of oral irrigation device and its solution type on the surface roughness and topography of Bulk-fill composite resins

**DOI:** 10.4317/jced.59004

**Published:** 2022-02-01

**Authors:** Fereshteh Naser-Alavi, Ashkan Salari, Niloofar Moein, Ainaz Talebzadeh

**Affiliations:** 1 Assistant professor, Dental Sciences Research Center, Department of Operative Dentistry, School of Dentistry, Guilan University of Medical Sciences, Rasht, Iran; 2 Assistant professor, Dental Sciences Research Center, Department of Periodontics, School of Dentistry, Guilan University of Medical Sciences, Rasht, Iran; 3 Assistant professor, Department of Operative Dentistry, School of Dentistry, Guilan University of Medical Sciences, Rasht, Iran; 4 Student of dentistry, School of Dentistry, Inter national branch of Guilan University of Medical Science, Anzali, Iran

## Abstract

**Background:**

Surface roughness and topography of composite resin materials have a significant role in biofilm aggregation, periodontitis, and recurrent caries. The present study evaluated the effect of the Waterpik oral irrigation device (OID) with different solution [water/ chlorhexidine (CHX)] on the surface roughness and topography of microhybrid (x-tra fil) and nanohybrid (Tetric N-Ceram Bulk) bulk-fill composite resins.

**Material and Methods:**

Disk-shaped samples were prepared from each composite resin, measuring 5 mm in diameter and 3 mm in height, and assigned to three groups in terms of treatment (n=19): group A, control (storage in distilled water); group B, OID with water; group C, OID with 0.5% CHX. The samples were treated for eight weeks, simulating one-year use of OID. Profilometry and scanning electron microscopy (SEM) were used to evaluate and compare quantitatively surface roughness (Ra) and qualitative topography of composite resin surfaces before and after treatment. The data were analyzed with paired-samples, Wilcoxon, and generalized estimating equations tests (α=0.05).

**Results:**

The application of OID increased the Ra of composite resin compared to the control group (*P*<0.001). No significant difference was detected between the two solutions (water and CHX) (P=0.615). The effect of composite resin type and the cumulative effect of composite resin type and OID solution were not significant on the surface roughness changes of the samples (*P*=0.243 and *P*=0.464, respectively).

**Conclusions:**

OID with water and CHX solutions increased the surface roughness and topographic changes of microhybrid and nanohybrid bulk-fill composite resins.

** Key words:**Composite resins, irrigation, mouthrinse, surface roughness, topography.

## Introduction

Improvements in mechanical, surface, optical properties and the clinical performance of composite resins have led to the introduction of a wide variety of these restorative materials. Recently, bulk-fill composite resins with wide variations and different applications have been introduced to the dental field, available in two forms of flowable and high viscosity. The advantages of these materials include improvements in polymerization depth, decreased polymerization shrinkage stress and working time compared to conventional composite resins (1,2).

Surface roughness is among the most important properties of restorative materials. Composite resins have a wide variety of fillers. Any change in the structure and composition of materials might affect their surface roughness and topography. Surface roughness of a restorative material has a significant role in its esthetic appearance and biological success. Rough surfaces have unfavorable optical properties and are more susceptible to staining and retention of plaque and bacteria (3). In addition, a smooth composite resin surface brings about more comfort for the patient; a 0.25-0.5 µm increase in surface roughness might be detected by the patient’s tongue tip, resulting in patient discomfort (4). The wear resistance of composite resins depends on the resin matrix, the filler shape and size, and the quality of polymerization. The smoothness and luster of composite resin materials in the oral cavity might be compromised over time by various factors, including temperature, pH, moisture of the liquids contacting the resin materials, and oral hygiene methods (5-7).

The use of mechanical oral hygiene methods, including toothbrushing and flossing, is the main focus of dentists in controlling microbial plaque. However, in the oral cavity, the mechanical methods of plaque control alone are not adequate for maintaining gingival health and preventing dental caries and periodontal diseases due to the lack of access to some areas. Antimicrobial mouthwashes increase the efficacy of mechanical methods and prevent gingival and dental diseases (8,9). Chlorhexidine digluconate has a long history of inhibiting dental plaque and is considered the gold standard in decreasing microbial counts. Its lower concentrations (0.05%) can be used daily for a long time with minimum side effects. One of the problems with mouthwashes is a lack of proper penetration to inaccessible areas such as interproximal gingival areas and periodontal pockets (10).

Recently, the use of an oral irrigation device (OID), also called water-jet and water flosser, has increased as an auxiliary device for oral hygiene and removing microbial plaque, especially in areas that are less accessible to toothbrushes. The mechanism of action of this device relies on irrigation through pulsation and high water pressure. The applied water pressure produces a hydraulic shearing force that can remove bacterial biofilms from the areas under treatment. When a water-jet device is used at home, a minimum pressure of 60 PSI and a pulse rate of 1200-1400 are required for clinical efficacy. The use of higher pressures with this device is also safe (11). Scanning electron microscopy (SEM) evaluations have shown that a 3-second use on any surface can eliminate 99.9% of biofilms on that surface (12). The device can be used several times a day depending on the patient’s needs, especially in patients with gingival problems or those who have problems with toothbrushing and flossing. This oral irrigation device can be used with different solutions, including water and antiseptics or antibacterial mouthwashes. A diluted solution of CHX can be used with a water-jet; 0.04% and 0.06% concentrations of CHX have proved effective in clinical studies (13). It has been reported to be safe concerning the effect of water pressure on gingival and junctional epithelium attachments (14). The main concern with the use of this device is the possible effect of pressure and type of the irrigation solution on increasing surface roughness and the loss of the luster of polymer-based restorative materials (i.e., composite resins) since these materials exhibit less hardness than the tooth enamel and have a heterogeneous structure (15).

Previous studies have evaluated the effect of toothbrushing and mouthwashes on the surface roughness of composite resins, reporting discrepancies in terms of the mouthwash and composite resin types (16-18). Alharbi *et al*. (15) evaluated the water-jet device on the surface roughness of different composite resin materials, in which an increase in the pressure of the device significantly increased surface roughness in composite resins with spherical fillers (Estelite Sigma Quick, Ceramic Sphere) compared to other composite resins.

The surface topography of composite resin materials has a significant role in biofilm aggregation and can increase the risk of periodontitis and recurrent caries (19). Adequate information is not available on the possible effect of oral irrigation devices and their solutions on surface changes of composite resins. Since oral hygiene auxiliary tools might affect oral health and the longevity of composite resin restorations, the present *in vitro* study aimed to evaluate the effect of two solutions with the oral irrigation device (i.e., water and 0.05% CHX) on the surface roughness and topography of microhybrid and nanohybrid high-viscosity bulk-fill composite resins.

## Material and Methods

The study protocol was approved by the scientific committee of Gulian University of Medical Sciences (IR.GUMS.REC.1399.363).

Sample size determination: Based on the pilot study and determining the effect of 0.3 (medium effect based on Cohen classification), power of 0.80, error level of 0.05 and 6 groups, a total sample size was obtained 111 which was considered 19 samples for each group.

In the present *in vitro* study, Tetric N-Ceram Bulk fill (TNCB) and x-tra fil (XTF) bulk-fill composite resins were used. Table 1 presents the characteristics of the composite resins used. Fifty-seven disk-shaped composite resin samples from each composite resin were prepared using a round silicone mold measuring 5 mm in diameter and 3 mm in height. To this end, the circle mold was placed on a glass slab and filled with composite resin. A Mylar matrix band was placed on the composite resin surface and pressed with another glass slab with a 500-g force for 30 seconds to achieve a smooth and homogenous surface without any porosity. Then the glass slab was removed, and the composite resins were light-cured through the Mylar matrix band to prevent the formation of the oxygen-inhibited layer (20). Each sample was cured with a polywave LED light-curing unit (X-cure, Woodpecker, Medical Instrument, Guangxi, China) at a light intensity of 1200 mw/cm2 (soft mode) for 20 seconds according to the manufacturer’s instructions. The tip of the probe (measuring 8 mm in diameter) was placed at a right angle to the material surface, barely touching it. The output of the light-curing device was checked with a radiometer (Woodpecker, Guangxi, China). The underneath of each sample was marked to make a distinction between the upper and lower surfaces of the samples. Then the samples were stored in distilled water at 37ºC for 24 hours. The samples were finished and polished with polishing disks (Sof-lex, 3MSPE, USA) from medium to fine to superfine, respectively. Each disk was used for 30 seconds parallel to the surface. After each polishing step, the sample surface was rinsed completely and dried with an air syringe, followed by polishing with the next polishing disk. Then the samples of each composite resin were randomly assigned to three subgroups (n=19).

**Table 1:**
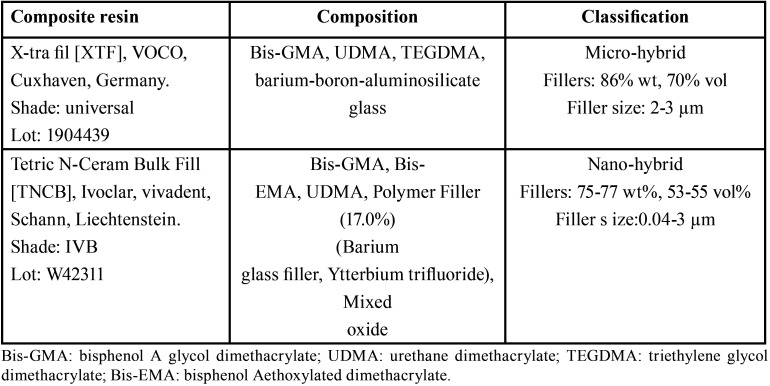
The characteristics of composite resin materials used in the study.

A. Control: storage in distilled water at ambient temperature

B. OID with water

C. OID with 0.05% CHX

To prepare 0.05% CHX solution for use with OID, 0.2% alcohol-free CHX (Vi-One, Tabriz, Iran) was used at a 1:3 ratio (mouthwash-to-water). The pH of the solution was measured with a pH meter (3505 JENWAY) before its application, which was around 6.2.

The samples of groups B and C were treated with the OID once a week for 8 weeks to prolong the process and better simulate the clinical condition. In each use of the OID, it was used for 5 minutes (totaling 40 minutes in 8 weeks). The estimated time of OID use was equal to the one year use of the device twice a day for 3 seconds each time on the surface. The classic jet tip of Waterpik WP100 (Waterpik, Inc, Fort Collins, USA) was used, which was suiTable for supragingival use. The tip of the device was placed at a right angle to the composite resin surface (according to the manufacturer’s instructions) at a distance of about 2 mm. The samples and the handle of the device were mounted and fixed to create a uniform condition. The device pressure was adjusted at #7, which is almost equal to 63 PSI. The liquid reservoir of the device was continuously replenished with the relevant solution so that it could be used continuously until the end of treatment. Then, each sample was rinsed with water spray for 10 seconds and stored in distilled water at room temperature until the next stage of treatment. The samples in group A (control) in both composite resins did not undergo any treatment and were stored in distilled water at room temperature. The distilled water was renewed in all the groups every day.

-Quantitative evaluation of surface roughness with a profilometer

A profilometer (Hommel Tester T8000) was used at a tracing length of 4 mm, a cutoff of 0.8 mm, and stylus speed of 0.5 mm/s to evaluate changes in the mean surface roughness of the samples at baseline (after polishing and before treatment) and after the treatment period in all groups. The surface roughness (Ra) of each sample was determined in µm at the center and two other points 1 mm away from the center, and their mean was calculated.

-Qualitative observation of surface topography using scanning electron microscopy (SEM)

**Figure 1:**
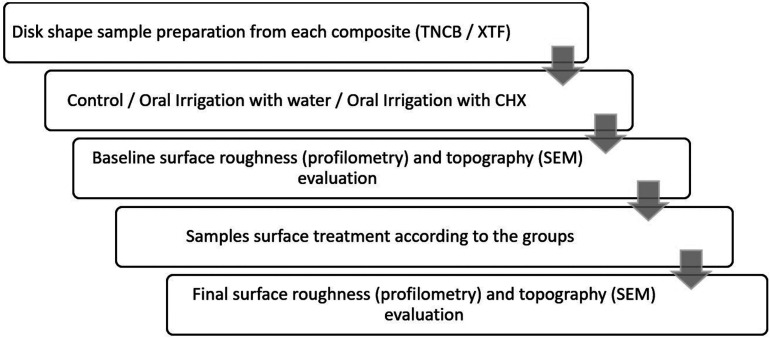
Diagram of the study process.

Two additional samples of each group were prepared for microscopic evaluation. Before and after the study procedures, Samples were sputter-coated with gold in a vacuum evaporator and evaluated with SEM (Mira//LMU, TESCAN, Czech Republic). The relevant micrographs were prepared at ×1000 and ×3000 magnifications, and each sample’s surface quality was evaluated and reported in detail. Figure 1 shows the methodology process of the present study.

-Data analysis

Data normality was analyzed with Shapiro-Wilk test, and variance equality was analyzed with Levene’s test after calculating the means and standard deviations of the samples’ surface roughness before and after treatment. In cases where the variances were equal, a pared-samples t-test was used to evaluate changes in surface roughness in each group; otherwise, the Wilcoxon test was used. The generalized estimating equations test was used to evaluate the effect of different factors on changes in surface roughness. SPSS 26 was used for statistical analyses at a significance level of *P*<0.05.

## Results

-Quantitative analysis of surface roughness with a profilometer

Table 2 presents the means and standard deviations of Ra in different groups. According to statistical analyses, Ra significantly increased in the groups treated with OID compared to the baseline. However, changes in the control groups were not significant (Table 2). According to the generalized estimating equations test, the surface roughness of the composite resins increased by a mean of 0.124 µm after treatment (*P*<0.001). The mean surface roughness in the control subgroups was significantly less than that in the OID groups in both composite resins (*P*<0.001), with no significant difference between the water and CHX groups (*P*=0.615). The effect of composite resin type and their cumulative effect with the oral irrigation solution type on surface roughness changes were not significant (*P*=0.243 and *P*=0.464, respectively) (Table 3).

**Table 2:**
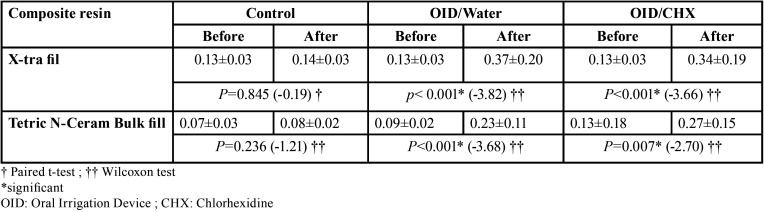
The means ± standard deviations of surface roughness (µm) in terms of composite resin and treatment types before and after treatment.

**Table 3:**
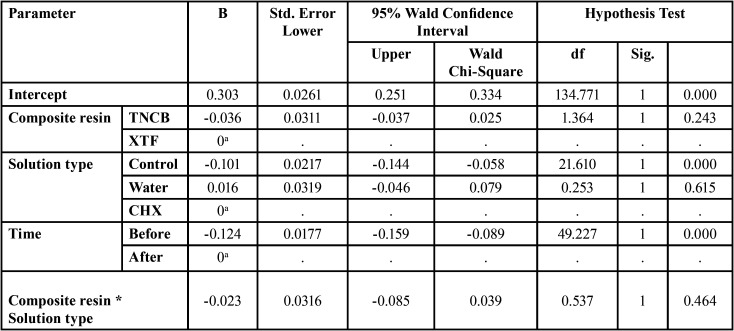
The effects of the factors composite resin, OID, and its solution on surface roughness changes (µm).

-Analysis of surface topography with SEM 

**Figure 2:**
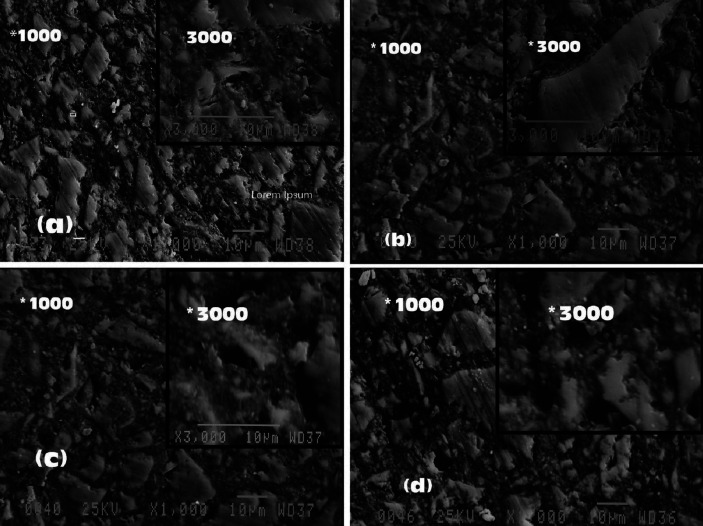
SEM emages of X-tra fil composite resin (*1000, *3000): (a) baseline, (b) control after 8 weeks, (c) treated with OID/water and (d) treated with OID/CHX. OID: Oral Irrigation Device; CHX: Chlorhexidine.

**Figure 3:**
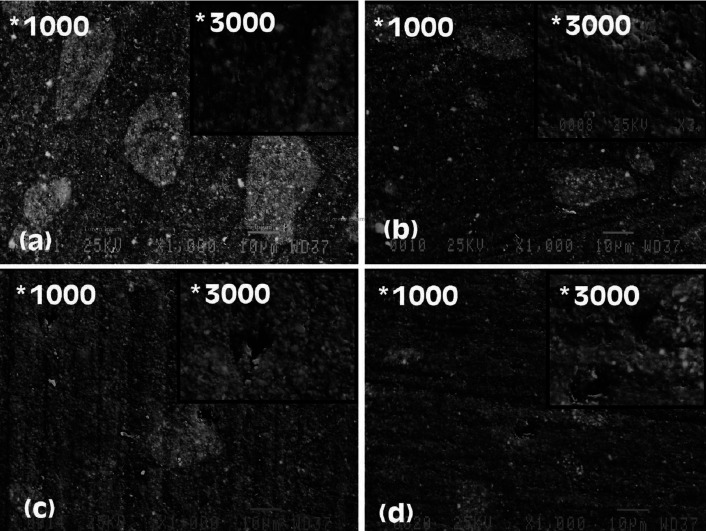
SEM emages of Tetric-N-Ceram Bulk composite resin (×1000, ×3000): (a) baseline, (b) control after 8 weeks, (c) treated with OID/water and (d) treated with OID/CHX. OID: Oral Irrigation Device; CHX: Chlorhexidine.

Initial SEM observations of the surface topography of composite resin samples showed no significant differences in the samples of the same material. The analysis of SEM images after treatment in the control groups showed no significant difference before and after treatment and an overall smoother surface than the other study groups. The oral irrigation device resulted in changes in surface morphology, increased surface roughness, and separation of fillers in both XTF and TNCB samples. It was not possible to make a distinction between the effects of different solutions of OID on the surface topography (Figs. 2,3).

## Discussion

Composite resin materials might undergo physical changes, including surface roughness changes, under the effect of mechanical and chemical mechanisms of oral hygiene measures. In this line, the material’s properties and the technique used are important (21-23). The surface characteristics of these materials are different depending on their composition and properties. Usually, the properties of filler particles, including their concentration, size, and shape, are the most important factors involved in wear resistance (24). The present study evaluated the effect of Waterpik WP 100 oral irrigation device with water and 0.05% CHX on the surface roughness and topography of microhybrid and nanohybrid bulk-fill composite resins. The results showed increased surface roughness of composite resin materials after applying OID with a 1-year use simulation. The composite resin type did not affect resistance to surface roughness changes. In the present study the device tip was placed at a right angle to the sample surface, and the device pressure gauge was set to #7 (almost equal to 63 PSI), according to a study by Jahn *et al*. (11), who reported that a minimum pressure of 60 PSI is necessary for the clinical efficacy of the device and proper removal of the dental plaque. The mechanism of action of OID relies on the high pressure of its solution. In this context, the shearing forces emanating from the device with its continuous application might result in the failure of the filler–matrix interface, separating fillers from the resin matrix, which is more probable in composite resins with large filler particles (15). In this line, hybrid composite resins exhibit poor performance and undergo greater wear. Larger filler particles of hybrid composite resins have more prominences on the material surface and serve as a cantilever, facilitating their separation from the resin matrix. However, in nanofilled and microfilled composite resins, smaller particles serve as a barrier against wear (25). It is possible that in the present study, larger filler particles in both XTF and TNCB hybrid composite resins have been separated from the resin matrix, creating large depressions or cavities on the surface and increasing the surface roughness during the re-evaluation of the surface with the profilometer. This can be explained by evaluating the SEM images of composite resin samples and the presence of large depressions on these images, indicating the separation of large filler particles. The results of the present study are somewhat consistent with a study by Alharbi *et al*. (15), in which the 50-PSI pressure of Aquarius OID did not change the surface roughness of composite resin materials, while the maximum pressure of the device (100 PSI) resulted in significant changes in the surface roughness of some composite resins. Microhybrid composite resins with spherical particles (Estelite Sigma Quik, Ceram Sphere) exhibited greater increases in surface roughness than other microhybrid composite reins (Z250, Tetric Evo-Ceram). It was reported that in addition to particle size, their shape, too, might affect the material’s wear resistance. The relative discrepancy in the present study results and the study above might be attributed to differences in the devices used, the procedural steps, the longer duration of the present study to simulate the aging and oral environment, and the difference in composite resin type. Bulk-fill composite resins use a new resin system, filler particles and a new silane. Changes in the structure and composition of materials can affect their surface wear resistance (18).

Previous studies have shown that the type of composite resin matrix, too, has an important role in its wear resistance. Therefore, composite resins with one class of fillers might exhibit differences in surface roughness changes. On the other hand, the classification of composite resins with nanotechnology structure (nanohybrid, nanomicrohybrid, microhybrid, and nanofilled) is carried out based on the manufacturer’s claims (26,27). Attention to these facts might help explain similar resistance of the composite resins evaluated in the present study. Therefore, attention to filler particle classification alone cannot successfully predict the material’s behavior in the face of wear.

Another finding of the present study was the similar effect of 0.05% CHX and water as the solutions used with OID on the surface roughness changes of both composite resins, i.e., CHX did not result in more changes in the surface roughness of resin materials than water. Consistent with this finding, in a study by Furtado and Amorim (17), immersion of bulk-fill composite resins in 0.05% CHX did not lead to surface changes in resin materials. However, 0.1% CHX concentration increased the surface roughness of these materials, which was attributed to the high concentration of CHX and its alcohol content. In a study by da Silva *et al*. (18), too, Listerine mouthwash with the highest alcohol content and the lowest pH than other mouthwashes caused the highest surface roughness in composite resin materials.

Alcohol is a bipolar molecule and results in the softening of the resin matrix and destruction of the polymer–filler interface (28). The ester groups of methacrylate monomers, too, can undergo hydrolysis by low pH values, making the composite resin material susceptible to erosion and abrasion (29). Therefore, surface changes of composite resin materials under the effect of mouthwashes are different, depending on their chemical composition and pH. The results of the present study can be justified by the use of 0.05% diluted CHX with no alcohol content and an approximate pH of 6.2.

In the present study, the quantitative measurements of surface roughness with a profilometer were mostly confirmed by analyzing SEM images. Based on the images, the filler particles were intact in the control groups with no treatment; however, large filler particles in both composite resins were separated from the matrix, leaving depressions and cavities on the surface after continuous application of OID with both solutions. The observations on the images were consistent with the increased surface roughness of the samples in measurements reported with the profilometer.

The application of OID in the present study increased the surface roughness of composite resins, slightly higher than the threshold for bacterial colonization [0.2 µm]; however, it was less than the threshold for clinical diagnosis by the patient’s tongue [0.5 µm] (4). Considering the limited data available at present, Waterpik OID is not entirely safe for all the composite resins. Continuous use of OID might lead to decreased properties and increased surface roughness of some hybrid composite resins. It is advisable to exercise caution in prescribing these auxiliary oral hygiene tools for patients with composite resin restorations in the cervical area of teeth, and their application should be supervised by a dentist to manage the possible effects, such as the re-polishing technique of the restoration.

One of the limitations of the present study was its *in vitro* nature. In the oral cavity, the saliva and pellicle on the restoration surfaces might affect the study results. In addition, considering the limited number of studies in this field, further studies are suggested with more diverse materials and clinical conditions.

## Conclusions

It might be concluded under the limitations of the present study that.

1. The use of Waterpik OID increased the surface roughness and led to changes in the surface topography of both hybrid bulk-fill composite resins.

2. The composite resin type did not affect resistance to surface changes.

3. CHX mouthwashes and water as oral irrigation solutions resulted in similar effects on the surface roughness of composite resins.
